# Cerebrovascular Disease and Depressive Symptomatology in Individuals With Subjective Cognitive Decline: A Community-Based Study

**DOI:** 10.3389/fnagi.2021.656990

**Published:** 2021-07-27

**Authors:** Patricia Diaz-Galvan, Nira Cedres, Nerea Figueroa, Jose Barroso, Eric Westman, Daniel Ferreira

**Affiliations:** ^1^Department of Neurobiology, Care Sciences, and Society, Division of Clinical Geriatrics, Center for Alzheimer Research, Karolinska Institutet (KI), Stockholm, Sweden; ^2^Department of Radiology, Mayo Clinic, Rochester, MN, United States; ^3^Department of Clinical Psychology, Psychobiology, and Methodology, Faculty of Psychology, University of La Laguna, San Cristóbal de La Laguna, Tenerife, Spain; ^4^Department of Neuroimaging, Center for Neuroimaging Sciences, Institute of Psychiatry, Psychology and Neuroscience, King's College London, London, United Kingdom

**Keywords:** subjective cognitive decline, subjective cognitive complaints, DTI, mean diffusivity, cerebrovascular disease, depressive symptomatology, mediation

## Abstract

Subjective cognitive decline (SCD) may be the first sign of Alzheimer's disease (AD), but it can also reflect other pathologies such as cerebrovascular disease or conditions like depressive symptomatology. The role of depressive symptomatology in SCD is controversial. We investigated the association between depressive symptomatology, cerebrovascular disease, and SCD. We recruited 225 cognitively unimpaired individuals from a prospective community-based study [mean age (SD) = 54.64 (10.18); age range 35–77 years; 55% women; 123 individuals with one or more subjective cognitive complaints, 102 individuals with zero complaints]. SCD was assessed with a scale of 9 memory and non-memory subjective complaints. Depressive symptomatology was assessed with established questionnaires. Cerebrovascular disease was assessed with magnetic resonance imaging markers of white matter signal abnormalities (WMSA) and mean diffusivity (MD). We combined correlation, multiple regression, and mediation analyses to investigate the association between depressive symptomatology, cerebrovascular disease, and SCD. We found that SCD was associated with more cerebrovascular disease, older age, and increased depressive symptomatology. In turn, depressive symptomatology was not associated with cerebrovascular disease. Variability in MD was mediated by WMSA burden, presumably reflecting cerebrovascular disease. We conclude that, in our community-based cohort, depressive symptomatology is associated with SCD but not with cerebrovascular disease. In addition, depressive symptomatology did not influence the association between cerebrovascular disease and SCD. We suggest that therapeutic interventions for depressive symptomatology could alleviate the psychological burden of negative emotions in people with SCD, and intervening on vascular risk factors to reduce cerebrovascular disease should be tested as an opportunity to minimize neurodegeneration in SCD individuals from the community.

## Introduction

It has been postulated that subjective cognitive decline (SCD) may be the first sign of Alzheimer's disease (AD) (Jessen et al., 2014). However, SCD has also been associated with other pathologies such as cerebrovascular disease (Diniz et al., 2013), especially in community-based studies (Slot et al., 2018). SCD has also been associated with other conditions like depressive symptomatology (Ginó et al., 2010; Zlatar et al., 2014; Cedres et al., 2019). Indeed, the role of depressive symptomatology in current diagnostic criteria of SCD is controversial (Jessen et al., 2014), and it is intensively discussed at the moment (Molinuevo et al., 2017; Rabin et al., 2017; Jessen et al., 2020).

Part of the discussion about the role of depressive symptomatology in SCD stems from the well-known association between depressive symptomatology and SCD (Clarnette et al., 2001; Reid and Maclullich, 2006; Ginó et al., 2010; Zlatar et al., 2014; Cedres et al., 2019). Due to this association, it was traditionally believed that SCD could merely reflect emotional factors (Apolinario et al., 2013; Yates et al., 2015; Burmester et al., 2016). However, there is convincing data showing that depressive symptomatology is a risk factor for future cognitive decline (Butters et al., 2008), or an early symptom of an underlying neurodegenerative disease (Alexopoulos et al., 2013). For example, late-life depression exacerbates the cognitive decline associated with both AD and cerebrovascular disease (Da Silva et al., 2013; Diniz et al., 2013). Also, cerebrovascular disease affects brain networks and causes early depressive symptoms (Murphy et al., 2007; Alexopoulos et al., 2013).

Cerebrovascular disease can be measured through markers assessed on magnetic resonance imaging (MRI) (Wardlaw et al., 2013). A common MRI marker of cerebrovascular disease is white matter signal abnormalities (WMSA), which can be assessed both on T1-weigthed images (white matter hypointensities) and T2-weigthed or fluid-attenuated inversion recovery (FLAIR) images (white matter hyperintensities). Another promising yet unspecific MRI marker is diffusion tensor imaging (DTI), which assesses microstructural alterations in the white matter that might be due to cerebrovascular disease (Zhou et al., 2008; Black et al., 2009; Kennedy and Raz, 2009; Salat et al., 2012). For example, DTI has been proposed as a marker to monitor the progression of cerebrovascular disease (Fu et al., 2012). Both WMSA and DTI alterations have been separately associated with depression (Murphy et al., 2007; Allan et al., 2016) and SCD (Wang et al., 2012; Selnes et al., 2013; Li et al., 2016; Cedres et al., 2019, 2020a; Ohlhauser et al., 2019). However, little is known about the association between cerebrovascular disease, depressive symptomatology, and SCD. This association is especially relevant in SCD individuals from the community, since the prevalence of cerebrovascular disease is significantly higher in community-based cohorts than in clinical cohorts of SCD individuals who seek medical help (Buckley et al., 2017; Slot et al., 2018).

**Figure 1:**
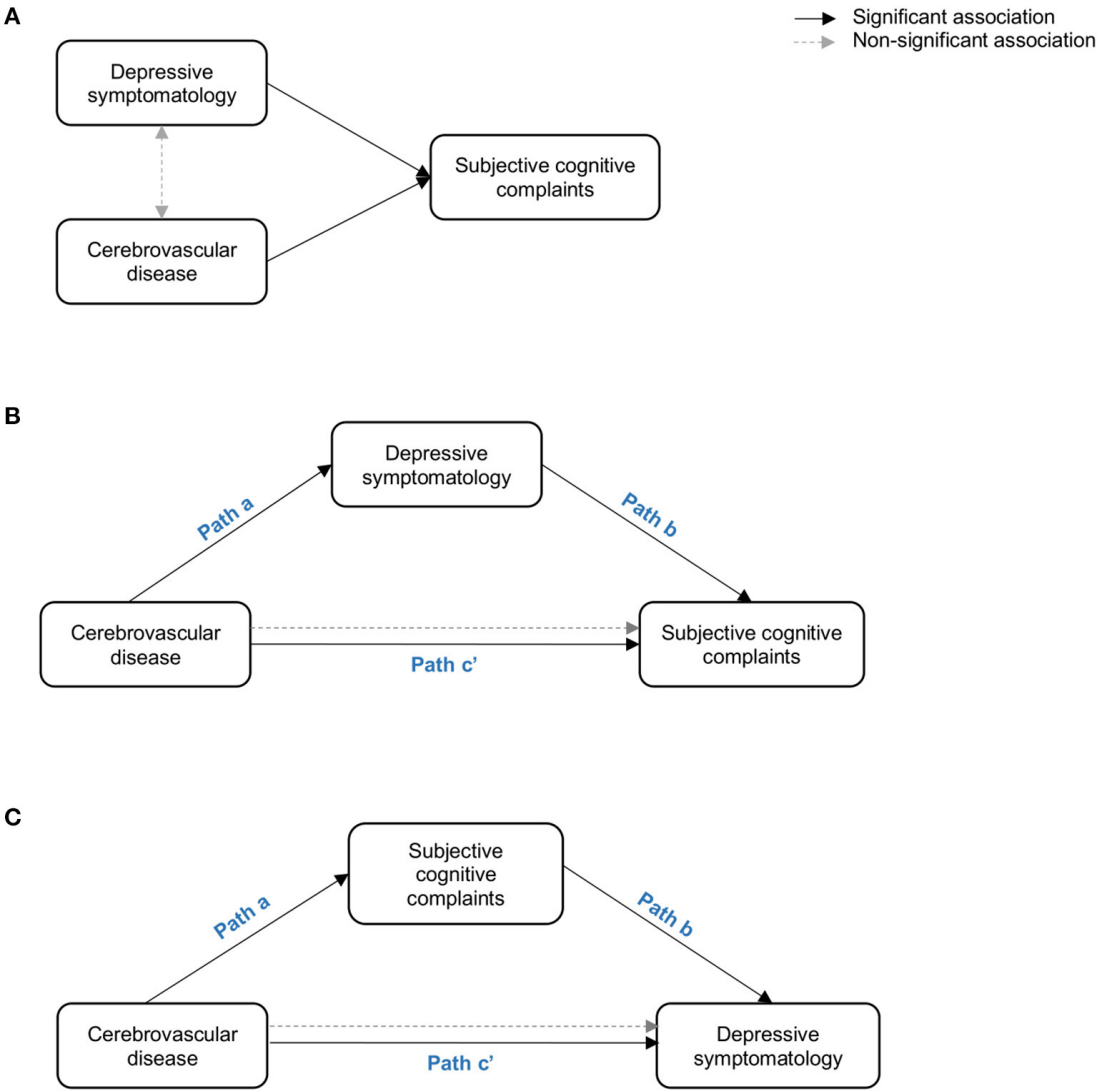
Representation of the three hypothetical scenarios: **(A)** depressive symptomatology co-exist with SCD (i.e. depressive symptomatology and cerebrovascular disease are independently associated with SCD; **(B)** depressive symptomatology influeces SCD by mediating the association between cerebrovascular disease and SCD; **(C)** depressive symptomatology reflects SCD (i.e. SCD mediates the association between cerebrovascular disease and depressive symptomatology. Paths in the figure refer to the associations betwen the independent variable, the mediator, and the dependent variable as described in Baron and Kenny' mediation framework (Baron and Kenny, 1986).

In keeping with the recent contribution from the international working group on SCD (Jessen et al., 2020), the role of depressive symptomatology in SCD still needs to be elucidated (Molinuevo et al., 2017; Rabin et al., 2017). Therefore, the first aim of this study was to investigate the role of depressive symptomatology in SCD in a community-based cohort. We hypothesized three possible scenarios where depressive symptomatology would (A) co-exist with SCD, (B) influence SCD, or (C) reflect SCD (Figure 1). We addressed these hypotheses by combining correlation, multiple regression, and mediation analyses. We wanted to: (A) prove that depressive symptomatology and cerebrovascular disease are independently associated with SCD, but there is no association between depressive symptomatology and cerebrovascular disease (hypothesis: depressive symptomatology co-exists with SCD); (B) depressive symptomatology is associated with cerebrovascular disease and it mediates the association between cerebrovascular disease and SCD (hypothesis: depressive symptomatology influences SCD by mediating the association between cerebrovascular disease and SCD); and (C) SCD mediates the association between cerebrovascular disease and depressive symptomatology (hypothesis: depressive symptomatology reflects SCD). The second aim of this study was to test the hypothesis that variability in the unspecific DTI marker of neurodegeneration would be associated with cerebrovascular disease in our community-based SCD cohort. In addition to correlation analysis, we also used mediation analysis to demonstrate that T1 WMSA burden would mediate the association between DTI abnormalities and SCD. Further, older individuals in our cohort have an increased WMSA burden (Nemy et al., 2020), a higher frequency of SCD (Cedres et al., 2019), and higher levels of depressive symptomatology (Machado et al., 2018). Hence, our third aim was to investigate the effect of aging in our analyses. We hypothesized that DTI abnormalities in SCD are associated with increased WMSA burden and older age.

## Methods

### Participants

A total of 225 cognitively unimpaired individuals from the GENIC-database (Machado et al., 2018) were included in the current study. The GENIC is a prospective community-based study from the Canary Islands (Spain). Recruitment was performed through primary care health centers, advertisements in local schools, and relatives, as well as acquaintances of the research staff. A more detailed description of the cohort is provided in previous publications (Ferreira et al., 2015; Machado et al., 2018; Gonzalez-Burgos et al., 2019).

All the individuals who received an MRI scan including both T1 and DTI sequences (see further down) were candidate cases for the current study. Inclusion criteria were in concordance with the SCD initiative (SCD-I) working group (Jessen et al., 2014): (1) Normal cognition, which was established in a two-step diagnostic procedure: Firstly, in a screening phase dementia was excluded based on altered activities of daily living and global cognition operationalized as a Blessed Rating Dementia Scale (BRDS) score >4 (Blessed et al., 1968), a Functional Activity Questionnaire (FAQ) score >5 (Pfeffer et al., 1982), and a Mini-Mental State Examination (MMSE) score <24 (Folstein et al., 1975); Secondly, mild cognitive impairment was excluded based on comprehensive neuropsychological assessment and age-, sex-, and education-adjusted normative data following current clinical criteria (Winblad et al., 2004). The neuropsychological protocol is summarized in the Supplementary Table 1. Briefly, the neuropsychological protocol was applied in two sessions and MRI scanning was conducted in a third session. In all participants, the time between neuropsychological assessment (first session) and MRI scanning was within 6 months (mean = 1.5 months, SD = 2.5); (2) No abnormal findings such as stroke, tumors, hippocampal sclerosis, etc., in MRI according to an experienced neuroradiologist; (3) no medical history of neurological and psychiatric disorders (including a diagnosis of major depression and/or individuals under treatment for depression), systemic diseases or head trauma; and (4) no history of substance abuse.

This study was approved by the ethics committee from the University of La Laguna (Spain). Participation was completely voluntarily, and all the individuals gave their written informed consent.

### Subjective Cognitive Decline

SCD was assessed with a questionnaire that covers subjective cognitive complaints (SCC) in different cognitive domains, including memory, orientation, executive functions, face recognition, language production, language comprehension, word-finding, reading and writing (Cedres et al., 2019). Participants answered nine yes/no questions referred to cognitive changes occurring during approximately the last 6 months. Answers were codified as 0 (absence of complaints) or 1 (presence of complaints). Answers were summed up and the total of complaints was obtained ranging from 0 to 9. In the current study, we use SCD when we refer to the clinical entity or concept of SCD, and we use SCC when we refer to the variable we used in our statistical analyses. The continuous variable of SCC was preferred to the dichotomous variable of SCD due to the nature of our statistical models and to avoid arbitrary clinical thresholds.

### Depressive Symptomatology

Depressive symptomatology was assessed with two validated scales. The Beck Depression Inventory (BDI, 21-items version) (Beck et al., 1961) was used for participants younger than 63 years of age, and the Geriatric Depression Scale (GDS, 15-items version) (Yesavage et al., 1982) was used for participants 63 years old or older. Following previous publications, BDI and GDS scores were transformed into z-scores and combined into one single variable for statistical analysis (BDI-GDS composite) (Ferreira et al., 2017; Cedres et al., 2019).

### MRI Data Acquisition and Image Processing

Participants were scanned using a 3.0T GE imaging system (General Electric, Milwaukee, WI, USA), located at the *Hospital Universitario de Canarias* in Tenerife, Spain. A three-dimensional T1-weighted Fast Spoiled Gradient Echo (FSPGR) sequence was acquired in sagittal plane: repetition time/echo time/inversion time = 8.73/1.74/650 ms., field of view = 250 × 250 mm, matrix = 250 × 250 mm, flip angle = 12°, slice thickness = 1 mm, voxel resolution = 1 × 1 × 1 mm. Also, a DTI sequence was acquired in axial plane: repetition time/echo time = 15.000/≈72 ms., field of view = 256 × 256 mm, matrix: 128 × 128 mm, directions = 31, *B*-value = 1,000, flip angle = 90°, slice thickness = 2.4 mm, voxel resolution = 2 × 2 × 2.4 mm. Full brain and skull coverage was required for the MRI datasets and detailed quality control was carried out on all MR images according to previously published criteria (Simmons et al., 2011).

T1-weighted images were processed and analyzed with the FreeSurfer 6.0.0 image analysis suite (http://surfer.nmr.mgh.harvard.edu/). The FreeSurfer measure of white matter hypointensities was used as a surrogate marker of cerebrovascular disease, and referred to as WMSA in the current study. Briefly, FreeSurfer uses a probabilistic procedure to detect hypointensities in the white matter and labels them as WMSA (Fischl et al., 2002). This procedure has previously demonstrated sensitivity in measuring white matter damage both in healthy individuals and in patients with Alzheimer's disease (Salat et al., 2010; Leritz et al., 2014). These T1-weighted WMSA correlate with hyperintensity volumes measured on T2/FLAIR, as well as with microstructural white matter changes as measured on diffusion tensor imaging data (Leritz et al., 2014; Riphagen et al., 2018; Cedres et al., 2020b; Nemy et al., 2020). The estimated total intracranial volume (TIV) was also obtained from FreeSurfer in order to adjust the WMSA volume by the TIV. This adjustment was performed by dividing the WMSA volume by the TIV of each individual (Voevodskaya, 2014). The TIV-adjusted WMSA measure was used for statistical analyses.

The DTI images were pre-processed and analyzed with the FSL software (http://www.fmrib.ox.ac.uk/fsl/index.html), using the FDT and tract-based spatial statistics (TBSS) tools. The mean diffusivity (MD) index was selected as our measure of interest in this study because MD has shown to be an earlier indicator of neurodegeneration compared to other diffusivity measures (Liu et al., 2013; Li et al., 2015).

All the data were processed through theHiveDB system (Muehlboeck et al., 2014). Careful visual quality control was performed on all output data obtained from both FreeSurfer and FSL, and manual edits were applied when needed.

### Statistical Analysis

The DTI data was analyzed through a voxel-based approach on the white matter skeleton, using the FSL software (Smith et al., 2006). Separate general linear models were fitted at the voxel level with MD as the dependent variable and SCC, depressive symptomatology, WMSA, or age as independent variables. Permutation-based non-parametric testing with 5,000 iterations was used followed by threshold-free cluster enhancement (TFCE) and the family-wise error (FWE) correction for multiple testing (*p* ≤ 0.01, two-sided). Average MD values of significant clusters in individual's native space were extracted as new measures for further analysis (e.g., SCC-related MD and WMSA-related MD, please see the results section below). In addition, the global MD was extracted as a measure of mean MD values in the whole white matter skeleton.

We designed an approach based on correlation, multiple regression, and mediation analyses to address our first aim: to investigate de role of the depressive symptomatology in SCD (Figure 1). Firstly, bivariate Pearson correlations were used to study relationships between SCC and depressive symptomatology, WMSA, and MD measures. Secondly, multiple linear regression models were used to further investigate partial associations of depressive symptomatology, WMSA, and MD measures (predictors) with SCC (outcome variable). Thirdly, these analyses were complemented with mediation models when the three basic conditions of mediation analysis were satisfied (Baron and Kenny, 1986): (1) there is a significant association between the mediator and the independent variable; (2) there is a significant association between the independent variable and the dependent variable; and (3) there is a significant association between the mediator and the dependent variable when the independent variable is also included in the model. For an illustration of the mediation models please see Figure 1.

Mediation analysis was also used to investigate our second aim: to investigate whether WMSA mediates the association between MD and SCC. Mediation was based on the average direct effect (ADE), the average causal mediation effect (ACME), and the total effect. Briefly, the ADE represents the direct effect of the independent variable on the dependent variable, while the ACME represents the indirect effect of the independent variable on the dependent variable, through the mediator variable. The total effect represents the sum of the ACME and the ADE. When the ACME is statistically significant (in conjunction with a significant total effect) there is a mediation effect that can be of two types: full mediation, when the ACME is significant but the ADE is non-significant; and partial mediation, when both the ACME and the ADE are significant (Tingley et al., 2014). The ACME and the ADE were calculated by using confidence intervals based on non-parametric bootstrap sampling (1,s000 simulations).

To address our third aim—to investigate the effect of aging in our analyses—we repeated the above-mentioned regression models including age as a covariate, and we tested for bivariate Pearson's correlations for age with SCC, depressive symptomatology, WMSA, and MD.

Statistical analyses were conducted using the R statistical software (http://www.r-project.org). A *p* < 0.05 (two-tailed) was deemed significant in all these analyses.

## Results

**Figure 2:**
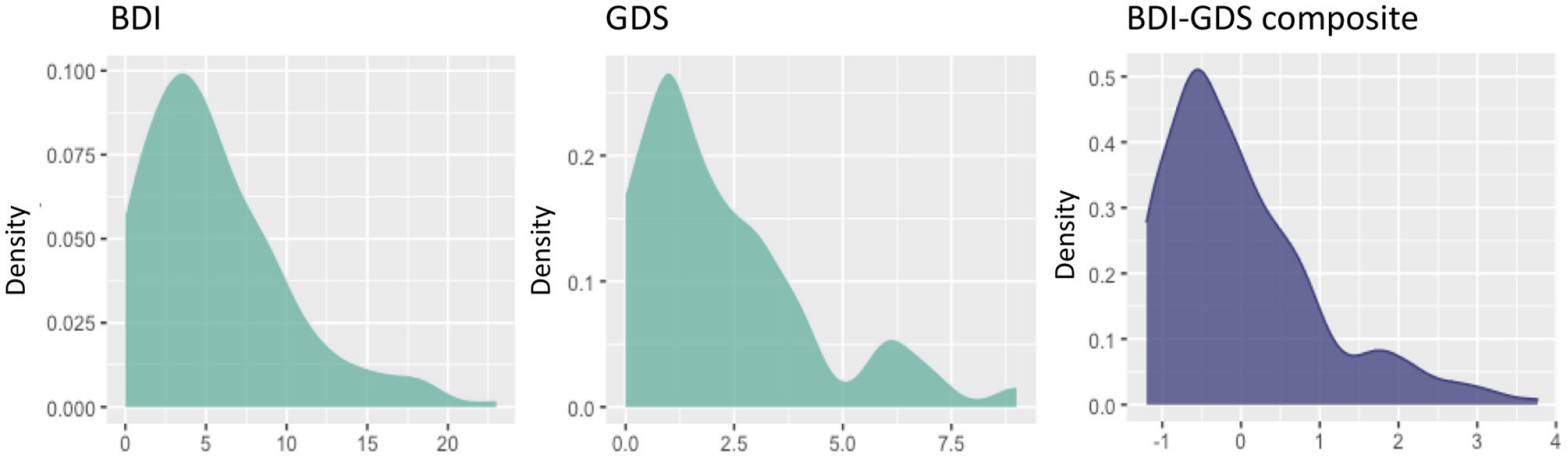
Distribution of the variables of depressive symptomatology. Scores on the x-axis (original scores from the BDI and the GDS, or z-scores from the BDI-GDS composite measure), and densities on the y-axis. BDI, Beck Depression Inventory; GDS, Geriatric Depression Scale.

The demographic and clinical characteristics of the cohort are described in Table 1. A total of 123 (55%) participants endorsed one or more SCC, while 102 (45%) participants reported zero SCC (number of complaints: mean = 0.92; SD = 1.1, range = 0–6). There were significantly more women in the subgroup of individuals with one or more SCC compared with those individuals with zero SCC (Table 1). Individuals with one or more SCC also showed significantly lower scores in the WAIS-III Information subtest after correcting for sex. Individuals with one or more SCC also had significantly lower scores in the MMSE; higher scores in the BDRS; and more depressive symptoms. These differences remained significant after controlling for the effect of sex and WAIS-III Information subtest. The proportion of individuals with high cholesterol and blood pressure was higher in the subgroup with one or more SCC than the group with zero SCC. Individuals with one or more SCC had a higher WMSA burden and worse white matter integrity (i.e., higher MD values) than individuals with zero SCC. Regarding depressive symptomatology irrespective of SCC, participants younger than 63 years scored between 0 and 23 in the BDI (mean = 5.6; SD = 4.6), and participants 63 years old or older scored between 0 and 9 in the GDS (mean = 2.3; SD = 2.1). The distributions of BDI, GDS, and the BDI-GDS composite variable are shown in Figure 2.

**Table 1:** Demographic and clinical characteristics.

	**Whole sample** **(*n* = 225)**	**Individuals with one or more SCC** **(*n* = 123)**	**Individuals with zero SCC** **(*n* = 102)**	***p***
Age	54.6 (10.2)	56.9 (11.0)	51.9 (8.3)	<0.001
Sex (% women)	55	64	43	0.002
Education level (% 0/1/2/3/4)^a^	0/3/35/25/37	4/42/26/29	2/28/25/45	0.07
Information (WAIS-III)	16.8 (6.0)	15.6 (6.0)	18.3 (5.7)	<0.001
MMSE	28.9 (1.2)	28.7 (1.3)	29.1 (1.0)	0.018
BDRS	0.6 (0.9)	0.7 (1.0)	0.4 (0.8)	0.017
FAQ	0.3 (0.7)	0.3 (0.6)	0.3 (0.8)	0.357
Subjective cognitive complaints^b^	0.9 (1.1)	1.7 (1.0)	0 (0)	–
Depressive symptomatology^c^	0 (1)	0.3 (1.0)	−0.3 (0.8)	<0.001
Cholesterol, n(%)	41 (18)	30 (73)	11 (27)	0.017
High blood pressure, n(%)	51 (23)	35 (69)	16 (31)	0.041
Diabetes, n(%)	5 (2)	4 (3)	1 (1)	0.507
Global MD^d^	7.4 (0.2)	7.5 (0.2)	7.4 (0.2)	0.018
WMSA volume	14.9 (13.1)	16.9 (15.6)	12.5 (8.7)	0.01

*Values correspond to the mean (standard deviation), except for Sex and Education level, in which values correspond to percentage (%). P-values correspond to results of group comparisons between individuals with one or more subjective cognitive complaints (SCC) and individuals with zero SCC*.

a *Education Level: illiterate (0); acquired reading and/or writing skills (1); primary level (2); secondary level (3); university level (4)*.

b *Subjective cognitive complaints were studied through nine yes/no questions as explained in the methods*.

c *Depressive symptomatology was estimated by transforming BDI and GDS scores into z scores and then combined them into one single variable*.

d *MD values were multiplied by 10,000. WAIS, Wechsler Adult Intelligence Scale; MMSE, Mini-Mental State Examination; BDRS, Blessed Dementia Rating Scale; FAQ, Functional Activity Questionnaire; BDI, Beck Depression Inventory; GDS, Geriatric Depression Scale; WMSA, White Matter Signal Abnormalities; MD, Mean Diffusivity*.

### First Aim: The Role of Depressive Symptomatology

**Figure 3:**
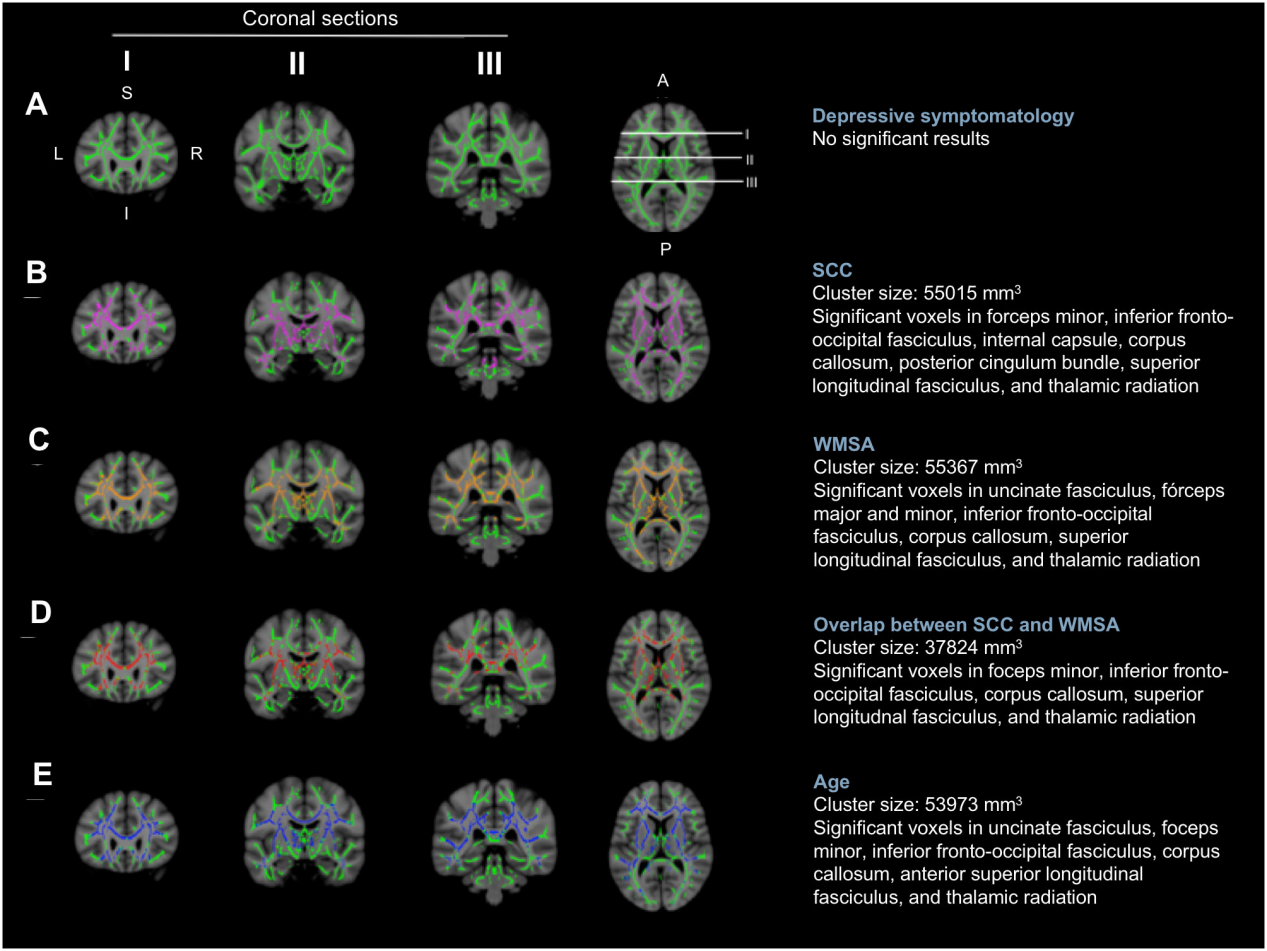
Voxel-wise correlations of MD values with depressive symptomatology, SCC, WMSA, and age. The white matter skeleton is depicted in green. Significant voxels are colored in pink [**(B)** the association between MD values and SCC], orange [**(C)** the association between MD values and WMSA], red [**(D)** overlap of the association between MD values and SCC, and MD values and WMSA], and blue [**(E)** the association between MD values and age]. No significant voxels were obtained for the association between MD values and depressive symptomatology **(A)**. L, left; R, right; S, superior; I, inferior; A, anterior; P, posterior; MD, mean diffusivity; SCC, subjective cognitive complaints; WMSA, white matter signal abnormalities; mm, millimeters.

The first aim of this study was to investigate the role of depressive symptomatology in SCD, under the hypotheses that depressive symptomatology would (A) co-exist with SCD, (B) influence SCD, or (C) reflect SCD (Figure 1). Correlation analyses showed that higher scores in depressive symptomatology were associated with a higher number of SCC (*r* = 0.340, *p* < 0.001). In contrast, depressive symptomatology did not correlate with the global MD (*r* = 0.076, *p* = 0.321) or WMSA (*r* = 0.003, *p* = 0.961). Depressive symptomatology did not correlate with MD values at the voxel level either (Figure 3A). Based on these results, we could not satisfy some of the three basic conditions for mediation analysis proposed by Baron and Kenny's (1986) (i.e., the association between depressive symptomatology and MD or WMSA). Hence, we could not test for mediation models including depressive symptomatology together with WMSA or global MD (Figures 1B,C). We next conducted a multiple linear regression model to investigate the partial association of depressive symptomatology, WMSA, and MD with SCC. We included SCC as the criterion variable, and WMSA, global MD, and depressive symptomatology as the predictors (Table 2; model 1). This model was significant [*F*_(3, 221)_ = 15.655, *p* < 0.001, *R*^2^ adj. = 0.16], indicating that depressive symptomatology and WMSA were independently associated with SCC. In contrast, global MD was not significant (Table 2). However, the lack of a significant effect for global MD may due to the fact that the measure of global MD may include areas that are not involved in SCC. Hence, in a second model, we restricted MD values to those voxels that were significantly related with SCC (“average SCC-related MD,” see below). We observed that depressive symptomatology continued to be a significant predictor of SCC, but WMSA was no longer a significant predictor when the average SCC-related MD was also in the model [Table 2; model 2, full model: *F*_(3, 221)_ = 20.987, *p* < 0.001, *R*^2^ adj. = 0.211]. In correlation analysis, depressive symptomatology was not correlated with the average SCC-related MD either (*r* = 0.117, *p* = 0.079).

**Table 2:** Partial association of depressive symptomatology, WMSA, MD, and age with SCC (multiple regression models).

	***R*^**2**^**	***B***	***SE B***	**β**	***p***
**Model 1**	**0.16**				**<0.001**
Depressive symptomatology		0.36	0.07	0.33	<0.001
WMSA		0.01	0.01	0.17	0.011
Global MD		0.63	0.33	0.13	0.059
**Model 2**	**0.21**				**<0.001**
Depressive symptomatology		0.34	0.07	0.31	<0.001
WMSA		0.01	0.01	0.08	0.271
Average SCC-related MD		0.12	0.03	0.28	<0.001
**Model 3**	**0.20**				**<0.001**
Depressive symptomatology		0.35	0.06	0.30	<0.001
Average SCC&WMSA-related MD		0.10	0.03	0.31	<0.001
***Including age as predictor***					
**Model 4**	**0.23**				**<0.001**
Depressive symptomatology		0.35	0.06	0.31	<0.001
WMSA		0.002	0.01	0.03	0.663
Global MD		0.28	0.33	0.06	0.391
Age		0.03	0.01	0.31	<0.001
**Model 5**	**0.25**				**<0.001**
Depressive symptomatology		0.33	0.06	0.30	<0.001
WMSA		−0.00	0.01	−0.01	0.90
Average SCC-related MD		0.08	0.03	0.19	0.012
Age		0.03	0.01	0.25	0.001
**Model 6**	**0.24**				**<0.001**
Depressive symptomatology		0.35	0.06	0.31	<0.001
Average SCC&WMSA-related MD		0.11	0.03	0.14	0.055
Age		0.03	0.01	0.27	<0.001

*Values correspond to R^2^ and its significance for each model. For each predictor in the models, values correspond to beta values (B) and their standard errors (SE B), as well as the standardized betas (β) and their significance values. BDI, Beck Depression Inventory; GDS, Geriatric Depression Scale; WMSA, White Matter Signal Abnormalities; MD, Mean Diffusivity; SCC, subjective cognitive complaints*.

These results suggest that depressive symptomatology may co-exist with SCC (Figure 1A). To fully prove that hypothesis we had to demonstrate that cerebrovascular disease is also associated with SCC. Hence, we conducted complementary analyses to further characterize the association of WMSA and MD with SCC. A higher burden of WMSA and a higher global MD correlated with a higher number of SCC (*r* = 0.216, *p* = 0.001, and *r* = 0.210, *p* = 0.002, respectively). The voxel-based analysis showed that the association between higher MD values and a higher number of SCC involved most of the white matter skeleton, with a tendency to spare the occipital white matter and the anterior part of the cingulum bundle (Figure 3B). The average MD value of these SCC related areas was extracted in a new variable (“average SCC-related MD”) for further analysis.

### Second Aim: The Contribution of WMSA to Variability in MD

The second aim of this study was to test the hypothesis that variability in MD, an unspecific DTI biomarker of neurodegeneration, would mediate cerebrovascular disease as measured by WMSA. Correlation analyses showed that a higher global MD correlated with a higher burden of WMSA (*r* = 0.370, *p* < 0.001), and a higher MD in areas specifically associated with SCC (“average SCC-related MD”) showed an even stronger correlation with a higher burden of WMSA (*r* = 0.492, *p* < 0.001). At the voxel level, the association between higher MD levels and higher WMSA burden involved most of the white matter skeleton, with a tendency to spare the internal capsule, the occipital white matter, and the cingulum bundle (Figure 3C). The average MD value of WMSA-related areas was extracted in a new variable (“average WMSA-related MD”) for further analysis. Results showed that the correlation coefficient of the association between SCC and the average WMSA-related MD (*r* = 0.267) was larger than the correlation coefficient of the association between SCC and global MD (*r* = 0.210). We also assessed the conjunction between the association of MD with SCC and WMSA. When we overlapped these two maps, MD values in forceps minor, corpus callosum, superior longitudinal fasciculus, inferior fronto-occipital fasciculus, and thalamic radiation were associated with both SCC and WMSA burden (Figure 3D). The measure “average SCC&WMSA-related MD” was calculated as the conjunction between these two maps. A new multiple regression model was conducted to investigate the partial association of depressive symptomatology and the MD voxels that were associated with both SCC and WMSA burden (“average SCC&WMSA-related MD”) with SCC. The model included depressive symptomatology and “average SCC&WMSA-related MD” as predictors, and SCC as the criterion (Table 2; model 3). This model was significant [*F*_(2, 222)_ = 28.534, *p* < 0.001, *R*^2^ adj. = 0.197], indicating that both the average SCC&WMSA-related MD (β = 0.300, *p* < 0.001) and depressive symptomatology (β = 0.309 *p* < 0.001) were independently associated with SCC.

Finally, we used mediation analysis to investigate whether WMSA mediates the association between global MD and SCC. We found that WMSA significantly mediated the association between global MD and SCC (ACME = 2960.825; *p* = 0.026). This mediation effect was partial because the direct effect of global MD on SCC was also significant (ADE = 7590.678, *p* = 0.032).

### Third Aim: The Effect of Aging

The third aim of this study was to investigate the effect of aging in our data. Correlation analyses showed that an older age correlated with a higher volume of WMSA (*r* = 0.521, *p* < 0.001), a higher global MD (*r* = 0.387, *p* < 0.001), a higher number of SCC (*r* = 0.373, *p* < 0.001), and higher scores in depressive symptomatology (*r* = 0.069, *p* = 0.030). The voxel-based analysis showed that the association between an older age and higher MD values involved most of the white matter skeleton, with a tendency to spare the occipital and parietal white matter and tracts going through the internal capsule and the cingulum bundle (Figure 3E). Next, we added age as an extra predictor to the multiple regression models reported for the first and second aims. The model for global MD (model 4 in Table 2) was significant [*F*_(4, 222)_ = 17.581, *p* < 0.001, *R*^2^ adj. = 0.228], showing that age was the main predictor of SCC, followed by depressive symptomatology. In contrast, WMSA and global MD were not significant as predictors. The model specific for MD areas involved in SCC (“average SCC-related MD,” model 5 in Table 2) was significant [*F*_(4, 220)_ = 19.466, *p* < 0.001, *R*^2^ adj. = 0.248], showing that age, depressive symptomatology, and the average SCC-related MD were significant predictors of SCC, while WMSA was not significant (*p* = 0.900). Finally, the model for the average SCC&WMSA-related MD (model 6 in Table 2) was significant [*F*_(3, 221)_ = 24.676, *p* < 0.001, *R*^2^ adj. = 0.241], showing that both depressive symptomatology and age (β = 0.268, *p* < 0.001) were independently associated with SCC, with a trend to significance for the average SCC&WMSA-related MD to predict SCC (β = 0.140, *p* = 0.055).

## Discussion

In this study, we tested the role of depressive symptomatology in the context of SCD and cerebrovascular disease using cross-sectional data from a community-based cohort. We also investigated whether DTI abnormalities (increased MD values) in SCD are associated with increased WMSA burden and older age. We operationalized SCD following the diagnostic criteria of the international working group on SCD (Jessen et al., 2014), and used the number of SCC (i.e., subjective cognitive complaints) in our statistical analyses. While our results showed that an increased depressive symptomatology is significantly associated with more SCC, we could not find a significant association of depressive symptomatology with WMSA or MD measures. We thus accepted the hypothesis of depressive symptomatology co-existing with SCC, independently of any MRI marker of cerebrovascular disease. In addition, we demonstrated that WMSA mediated the association between MD and SCC, and age had an important contribution to our findings.

The role of depressive symptomatology in SCD is controversial. While major depression is an exclusion criterion in current diagnostic criteria of SCD (Jessen et al., 2014), individual symptoms of depression that do not reach the threshold of a disorder are not considered a criterion for exclusion (Jessen et al., 2014). However, it is not clear what should be the exact threshold to exclude depression and how this type of symptomatology should be assessed in SCD (e.g., concurrent, past, subclinical, etc.). Currently, the most urgent need is to elucidate the role of subthreshold depressive symptomatology in SCD (Molinuevo et al., 2017; Jessen et al., 2020). In our relatively large community-based cohort, we approached this question by assessing subthreshold variability in depressive symptomatology in SCD individuals who did not have a diagnosis of major depression nor were under treatment for depression. Our analyses confirmed the well-known association between depressive symptomatology and SCD (Donovan et al., 2014, 2015; Buckley et al., 2016; Burmester et al., 2016; Lebedeva et al., 2018; Cedres et al., 2019), showing that a higher number of SCC was associated with increased depressive symptomatology in our cohort. In contrast, we could not demonstrate that an increased depressive symptomatology in our cohort is related with an older age, which would reflect the concept of late life depression (Diniz et al., 2013). In addition, we did not find an association of WMSA or MD measures with depressive symptomatology, as it would be predicted by the vascular depression hypothesis (Alexopoulos et al., 2013; Taylor et al., 2013). Altogether, our results suggest that the variability in depressive symptomatology in our cohort may be related to emotional factors rather than cerebrovascular disease or age-related factors. Moreover, despite its strong association with SCC, depressive symptomatology seems to just co-exist with SCD in our cohort, without influencing associations of SCC with markers of cerebrovascular disease or, as demonstrated in previous studies using the same cohort, with markers of gray matter degeneration (Cedres et al., 2019, 2020a) or clinical-cognitive status (Diaz-Galvan et al., 2021). Hence, SCD in our cohort does not seem to merely reflect emotional factors, as traditionally postulated (Apolinario et al., 2013; Yates et al., 2015; Burmester et al., 2016), but may rather reflect neurodegeneration and subclinical cognitive decline.

We observed a strong association of SCC with both WMSA and MD. Since WMSA correlated with MD, and WMSA mediated the association between MD and SCC, we suggest that variability in our MD measure may be influenced by cerebrovascular disease. In other words, despite being an unspecific marker, our MD measure may be reflecting cerebrovascular disease in our study. Other studies also highlighted the contribution of non-AD pathologies such as cerebrovascular disease to SCD in community-based cohorts (Diniz et al., 2013). The novelty of our study is the use of DTI to investigate white matter neurodegeneration associated with cerebrovascular disease, and the analysis of its topographical distribution. The utility of DTI measures as markers of cerebrovascular disease has previously been noted (Zhou et al., 2008; Black et al., 2009; Fu et al., 2012; Salat et al., 2012). Interestingly, in our study, the association between WMSA and MD in areas related with SCC (“average SCC-related MD”) was stronger than the association between WMSA and MD in the whole white matter skeleton (“global MD”). This suggests that the brain areas in which integrity of the white matter is associated with SCC seems to be more vulnerable to the effect of cerebrovascular disease than other white matter areas. Hence, cerebrovascular disease may be a contributor to SCC in our community-based cohort. This interpretation was further supported by our result showing that the association between SCC and MD in areas related with WMSA (“average WMSA-related MD”) was stronger than the association between SCC and MD in the whole white matter skeleton (“global MD”). Another important observation is that our associations between SCC and markers of cerebrovascular disease were related with or accounted by the age, as discussed further down.

We demonstrated the strong association between an older age and increased SCC, a finding that is well-established in the SCD literature (Derouesné et al., 1993; Wang et al., 2004; Jessen et al., 2010; van Harten et al., 2018; Cedres et al., 2019). We also demonstrated the strong association between an older age and increased cerebrovascular disease, in line with previous reports (Raz et al., 2012; Habes et al., 2016; Nemy et al., 2020). Whether this cerebrovascular disease in cognitively unimpaired older individuals indicates preclinical stages of vascular cognitive impairment or is rather a feature of normal aging when not reaching the clinical threshold is currently not known. The hypothesis of a preclinical stage is attractive in the context of our study, highlighting the capacity of SCD to reflect underlying neurodegenerative processes of presumably vascular origin. In a recent study using the same cohort we demonstrated that the effect of WMSA on cholinergic white matter pathways goes beyond the effect of age (Nemy et al., 2020). In our current study, including the age in our models removed the predictive partial effect of global MD, but the effect of MD in white matter areas associated with SCC remained significant. This suggests that while the integrity of the white matter overall seems to be primarily driven by increasing age, variability in the integrity of areas specific to SCC goes beyond the effect of aging (and depressive symptomatology). This finding was attenuated when investigating the integrity of areas specific to both SCC and WMSA (*p* = 0.055), suggesting that the cerebrovascular component that is related to SCC in our cohort may primarily be explained by increasing age, and not to stroke or other major vascular disease. This interpretation is congruent with the current definition of SCD, where neurological diseases other than AD are a criterion for exclusion (Jessen et al., 2014).

This study has some limitations. Although we did not find a significant association between depressive symptomatology and MRI markers of cerebrovascular disease, we cannot exclude that depressive symptomatology in our cohort could be an early symptom of other brain pathologies previously reported in SCD, such as amyloid-beta or tau pathologies (Amariglio et al., 2012; Perrotin et al., 2012; Buckley et al., 2017). The lack of biomarkers for amyloid-beta and tau pathologies is thus a limitation of our study. Nonetheless, vascular risk factors are highly prevalent in community-based cohorts like the one used in our study (Buckley et al., 2017), while AD pathologies are more prevalent in clinical cohorts (Kern et al., 2018). In the same vein, variability in MD was strongly associated with WMSA, but other pathologies such as amyloid-beta and tau could also be contributing to MD. This interpretation is supported by our finding of SCC-related MD remaining in our model, while WMSA was automatically removed when including the age in the same model. We believe that SCC-related MD reflects neurodegeneration beyond that related with an older age or a higher WMSA burden. However, our measure of WMSA is global, and it would be interesting to investigate WMSA intersecting SCC-related white matter tracts in future studies. We partially circumvented this by investigating the conjunction between WMSA-related MD and SCC-related MD. We reported the frequency of some vascular risk factors but a more complete characterization of vascular risk factors, as well as their contribution to our current findings is warranted in the future. In any case, these limitations do not compromise our main interpretation of depressive symptomatology as an emotional factor co-existing but not related with neurodegenerative factors that underlie SCC, since MD and WMSA correlated with SCC but not with depressive symptomatology. We used correlation, regression, and mediation analyses to investigate associations on cross-sectional data. Longitudinal designs or clinical trials to demonstrate that treating emotional or vascular risk factors reduces SCD would help to support potential causality in our current associations. Finally, previous studies showed that individuals with SCD differed in the frequency of affective symptoms and underlying neurodegeneration depending on whether they are recruited in the community or in clinical settings (Perrotin et al., 2017; Slot et al., 2018). Therefore, our current findings could also be tested in clinical-based samples, including SCD individuals who seek medical help.

In conclusion, depressive symptomatology co-exists with SCD and reflects emotional factors but not cerebrovascular disease, in our community-based cohort. In addition, we did not find any evidence for depressive symptomatology to influence the association between cerebrovascular disease and SCD. In our cohort, SCD reflected white matter neurodegeneration in spite of its association with depressive symptomatology. This highlights the clinical usefulness of SCD, especially in older individuals who often show subjective complaints, depressive symptomatology, and positive cerebrovascular disease biomarkers. A remark is that although SCD increased with age in our cohort, the association between white matter abnormalities and SCD was beyond the effect of aging. Therapeutic interventions for depressive symptomatology could alleviate the psychological burden of negative emotions in people SCD, and intervening on vascular risk factors to reduce cerebrovascular disease should be tested as an opportunity to minimize neurodegeneration in SCD individuals from the community. Another important contribution of the current study is the data reported to help understanding the association between cerebrovascular disease, depressive symptomatology, and SCD.

## Data Availability Statement

The raw data supporting the conclusions of this article will be available upon reasonable request from qualified researchers.

## Ethics Statement

The studies involving human participants were reviewed and approved by the ethics committee from the University of La Laguna (Spain). The patients/participants provided their written informed consent to participate in this study.

## Author Contributions

PD-G: data acquisitions, interpretation of results, writing of portions of the manuscript, and preparing figures. NC: data acquisitions, analysis and interpretation of results, writing of portions of the manuscript, and preparing figures. NF: study concept and design, data acquisitions, analysis and interpretation of results, and writing of portions of the manuscript. JB: supervision of the project, revision of manuscript, and funding. EW: revision of manuscript and funding. DF: study concept and design, data acquisition, interpretation of results, writing of portions of the manuscript, supervision of the study, and funding. All authors contributed to the article and approved the submitted version.

## Conflict of Interest

The authors declare that the research was conducted in the absence of any commercial or financial relationships that could be construed as a potential conflict of interest.

## Publisher's Note

All claims expressed in this article are solely those of the authors and do not necessarily represent those of their affiliated organizations, or those of the publisher, the editors and the reviewers. Any product that may be evaluated in this article, or claim that may be made by its manufacturer, is not guaranteed or endorsed by the publisher.

## Abbreviations

ACME: average causal mediation effect
AD: Alzheimer's disease
ADE: average direct effect
BDI: Beck's Depression Inventory
BDRS: Blessed Dementia Rating Scale
CVD: cerebrovascular disease
DTI: diffusion tensor imaging
FAQ: Functional Activity Questionnaire
FSPGR: Fast Spoiled Gradient Echo
GDS: Geriatric Depression Scale
GENIC: *Grupo de Estudios Neuropsicológicos de las Islas Canarias*
ICV: Intracranial volume
MD: Mean diffusivity
MMSE: Mini Mental State Examination
MRI: Structural magnetic resonance imaging
SCC: Subjective Cognitive Complaints
SCD: Subjective Cognitive Decline
SCD-I: Subjective Cognitive Decline initiative
TBSS: tract-based spatial statistics
WMSA: White matter signal abnormalities.
