# Supplementary material for: Cerebrovascular Disease and Depressive Symptomatology in Individuals With Subjective Cognitive Decline: A Community-Based Study
Source: Front Aging Neurosci. 2021 Jul 27;13:656990. doi: 10.3389/fnagi.2021.656990 (PMC8353130; doi:10.3389/fnagi.2021.656990)
Supplement: Supplementary file 1 [file Table_1.docx]

| **Supplementary Table 1. Neuropsychological protocol: description, measures, and references** | | |
| --- | --- | --- |
| **Cognitive functions – Neuropsychological test** | **Cognitive component** | **Measure** |
| ***Executive Functions*** | | |
| Controlled Oral Word Test (COWAT) (1) | Phonemic (letters) and semantic (animals) fluency | Number of correct words, perseverance and interference errors |
| Action fluency (2) | Actions fluency | Number of correct words, perseverance and interference errors |
| Luria’s Premotor Functions (Luria’s) (3) | Hand alternative movements, motor coordination and inhibition | Number of correct movements |
| ***Verbal and Visual Episodic Memory*** | | |
| Logical Memory (LM) (4) | Inmediate recall, delayed free recall (30 min.) and recognition task for each text (A and B) | Number of correct elements in each task |
| TAVEC (5) | Learning curve (5 trials), free inmediate and delay recall, after semantic clue inmediate and delay recall and recognition task | Number of correct words in each task and amount of different errors (perseverative and intrusión errors, false positive in recognition task) |
| 8/30 Spatial Recall Test (8/30). Modification of the 7/24 SRT (6) | Learning curve (5 trials), free inmediate and delay recall and recognition task | Number of correct elements in each task |
| Visual Reproduction (VR) (4) | Inmediate recall, delay recall and recognition task | Number of correct elements in each task |
| ***Visuocontructive, Visuoperceptive and Visuospacial*** | | |
| Visual Reproduction – Copy (4) | 2-D visuoconstructive abilities | Number of correct elements |
| Block Design (7) | 3-D visuocontructive abilities | Number of correct elements during an extended time (180 and 240 seconds for easy and difficult blocks respectively) |
| Facial Recognition Test – Short version (FRT) (8) | Visuoperceptive abilities | Number of correct items, max. 27 |
| Judgement of Line Orientation Test (JLOT) (8) | Visuospatial abilities | Number of correct items in first half (JLOT1) and second half (JLOT2), max. 15 |
| ***Language*** | | |
| Boston Naming Test (BNT) (9) | Lexical access by visual confrontation | Number of correct ítems, max 30, produced spontaniously and after a semantic clue |

**Supplementary references**

1. Benton A, Hamsher K, Sivan A. Multilingual aphasia examination. 2nd ed. Iowa City, IA: AJA Associates, University of Iowa; 1989.

2. Piatt AL, Fields JA, Paolo AM, Koller WC, Tr�ster AI. Lexical, semantic, and action verbal fluency in Parkinson’s disease with and without dementia. J Clin Exp Neuropsychol. 1999;21(4):435–43.

3. Christensen A. Luria’s neuropsychological investigation. 2nd ed. Copenhagen: Munksgaard; 1979.

4. Wechsler D. Wechsler Memory Scale. Technical Manual. 3rd ed. San Antonio: The Psychological Corporation; 1997 p.

5. Benedet M, Alejandre M. TAVEC: Test de Aprendizaje Verbal España- Complutense. Manual. Madrid: TEA Ediciones; 1998.

6. Rao S, Hammeke T, McQuillen M, Khatri B, Lloyd D. Memory disturbance in chronic progressive multiple sclerosis. *Arch Neurol*. 1984;41(6):625–31.

7. Wechsler D. Wechsler Adult Intelligence Scale - Administration and Scoring Manual. 3rd Editio. San Antonio, Texas: The Psychological Corporation; 1997.

8. Benton A, Hamsher S, Varney O, Spreen N. Contributions to neuropsychological assessment: A clinical manual. New York: Oxford University Press; 1983.

9. Kaplan E, Goodglass H, Weintraub S. Boston Naming Test. 2nd ed. Philadelphia: Lippincott Williams & Wilkins; 2001.
